# Comparative Evaluation of the Cariostatic and Antibacterial Efficacy of Nano Silver Fluoride With Silver Diamine Fluoride in Children With Special Health Care Needs: A Randomized Controlled Clinical Trial

**DOI:** 10.7759/cureus.86611

**Published:** 2025-06-23

**Authors:** Anija C. K, Nupur Ninawe, Shivani Sawant, Rashmi A Dongarwar, Anuradha V Khade, Hemraj Badhe

**Affiliations:** 1 Department of Pediatric and Preventive Dentistry, Government Dental College and Hospital, Nagpur, Nagpur, IND; 2 Department of Biotechnology, Rajiv Gandhi Biological Technology, Nagpur, IND; 3 Department of Pediatric Dentistry, Government Dental College and Hospital, Nagpur, Nagpur, IND

**Keywords:** antibacterial efficacy, caries arrest, cariostatic effect, nano silver fluoride, nsf, sdf, silver diamine fluoride, special children

## Abstract

Introduction

Dental caries remains a significant public health concern, particularly among children with special health care needs, who are at increased risk due to challenges in oral hygiene maintenance and limited access to dental care. Silver diamine fluoride (SDF) is widely used to arrest caries but is associated with undesirable black staining. Nano silver fluoride (NSF) has been introduced as a potential alternative, offering effective caries management without aesthetic drawbacks. Hence, this randomized controlled clinical trial aimed to evaluate and compare the cariostatic and antibacterial efficacy of NSF and SDF in children with special health care needs (CSHCN) aged 4-12 years.

Methods

Forty-two children with mild to moderate intellectual disability (IQ 40-70) and 3-4 active carious lesions were randomized into two groups: Group 1 received SDF and Group 2 received NSF. Saliva samples were collected at baseline and after one month to assess *Streptococcus mutans* and *Lactobacillus* counts. Clinical evaluation of caries arrest was performed at one, three, and six months.

Results

Both SDF and NSF significantly reduced the number of active carious lesions and salivary bacterial counts within groups (p < 0.001), with no statistically significant difference between groups at any interval (p > 0.05). NSF demonstrated comparable efficacy to SDF in both primary and permanent teeth. Notably, NSF did not cause black staining, offering a more aesthetic option, especially for anterior teeth.

Conclusion

NSF is an effective, safe, and visually favorable alternative to SDF for caries management in CSHCN.

## Introduction

Dental caries is defined as an “infectious microbiological disease of the teeth that results in localized dissolution and destruction of calcified tissues” [1]. It is a multifactorial condition, arising from the complex interplay of host factors, microbial agents, fermentable carbohydrates, and time [2]. The primary etiological agents responsible for dental caries are acidogenic and aciduric bacteria, predominantly *Streptococcus (S.) mutans *and *Lactobacilli*. These bacteria metabolize dietary sugars to produce organic acids, leading to a localized drop in pH. This acidic environment promotes demineralization of the tooth enamel, and if left unchecked, ultimately results in cavitation [3].

Among the diverse oral microbial flora, *Streptococcus mutans* plays a central role in the initiation of caries. This is attributed to its capacity to adhere to enamel surfaces via glucan-binding proteins, synthesize extracellular polysaccharides from sucrose using glucosyltransferases, and establish a cariogenic biofilm that favors acid accumulation [4]. These features allow S. mutans to persist even under low pH, initiating enamel demineralization. In contrast, *Lactobacilli* contribute more prominently to the progression of caries, as they thrive in acidic conditions and accelerate the breakdown of enamel and dentin [5]. Salivary counts of *S. mutans *have been shown to correlate positively with caries activity, reinforcing the importance of microbial control in caries prevention strategies [5,6].

Dental caries continues to be the most prevalent non-communicable disease globally, affecting individuals of all age groups. The World Health Organization (2022) reports that approximately 2.3 billion people suffer from caries in their permanent teeth, and more than 530 million children are affected in their primary dentition [7]. In India, schoolchildren exhibit high caries prevalence ranging from 64% to 78% in primary teeth and 18% to 67% in permanent teeth [8]. The burden is even more significant among children with special health care needs, where prevalence can reach up to 75.9%. This heightened susceptibility is largely due to challenges in maintaining oral hygiene, dietary factors, and limited access to preventive and therapeutic dental care. The management of dental caries in these children often necessitates sedation or general anesthesia due to behavioral and medical complexities [9].

Given these barriers, non-restorative caries treatments (NRCTs) have become increasingly important in managing dental caries, particularly in vulnerable populations [10]. One widely accepted NRCT is silver diamine fluoride (SDF), which has gained popularity for its dual ability to arrest caries progression and prevent new lesions. SDF comprises silver ions, fluoride ions, and ammonia. The silver ions exert antimicrobial effects by interacting with bacterial cell membranes, denaturing proteins, and interfering with DNA replication. Fluoride ions enhance remineralization by forming fluorapatite, a more acid-resistant mineral phase than hydroxyapatite, while ammonia stabilizes the solution's alkalinity and prolongs shelf life [11]. Additionally, SDF reacts with phosphate groups in hydroxyapatite to form silver phosphate and calcium fluoride, which seal the lesion surface and reduce lesion depth [12]. Numerous clinical studies support the biannual application of SDF as a safe, cost-effective method for caries arrest, particularly in pediatric and children with special health care needs populations [13].

However, despite its clinical benefits, SDF is not without drawbacks. One major concern is the permanent black staining of carious lesions, caused by the formation of silver oxide and silver sulfide, which can be aesthetically unappealing, especially in anterior teeth. Additionally, some patients report a transient metallic taste and minor gingival irritation upon application [14]. In response to these limitations, advances in nanotechnology have led to the development of nano silver fluoride (NSF), an alternative formulation for caries management.

NSF combines silver nanoparticles (AgNPs), fluoride, and stabilizing agents such as chitosan to achieve synergistic antibacterial and remineralizing effects. Silver nanoparticles exert antimicrobial action by disrupting bacterial membranes through oxidative stress and ion release, generating reactive oxygen species (ROS), and binding to bacterial enzymes and nucleic acids, which ultimately inhibit vital metabolic pathways and replication. Simultaneously, the fluoride component promotes remineralization by forming fluorapatite and enhancing enamel resistance to acid attacks [15]. Chitosan acts as both a stabilizer and an antimicrobial agent, increasing the overall efficacy and safety of NSF.

Recent NSF formulations have demonstrated superior antimicrobial efficacy without the black staining associated with SDF, making it a more aesthetically acceptable option for visible teeth. The smaller particle size of AgNPs enables deeper penetration into cariogenic biofilms, improving bacterial eradication and lesion arrest. Furthermore, NSF formulations have shown favorable physicochemical properties, such as long shelf stability (up to three years), eco-friendliness, and the need for only annual application, making them particularly well-suited for community-based dental programs and high-risk populations [10].

A study by Nagireddy et al. (2019) evaluated the caries-arresting potential of NSF in young children and found that 78% of decayed teeth showed arrest within seven days, and 72.91% remained arrested at five months [14]. These findings underscore NSF as a clinically effective, aesthetically favorable, and minimally invasive alternative to SDF for early childhood caries (ECC) management.

Despite these advancements, there remains a significant gap in the literature comparing the efficacy of NSF and SDF in children with special health care needs, a group at a particularly high risk of untreated dental caries. This study, therefore, aims to evaluate and compare the cariostatic and antibacterial effectiveness of nano silver fluoride versus silver diamine fluoride in children with special health care needs aged 4 to 12 years.

## Materials and methods

This study was planned as a parallel, two-arm, single-blinded, randomized controlled trial and conducted on children with special healthcare needs (CSHCN) (Mentally challenged, IQ score - 40 to 70 as per the Wechsler Intelligence Scale for Children III (WISC-III) classification of ages 4 to 12 years with 3 to 4 carious primary or permanent teeth (International Caries Detection and Assessment System (ICDAS) II score of 3 to 5) [16,17]. This study was conducted following ethical approval from the institutional ethical committee (IEC/09/14 dated: 23/03/2023) and was registered in the Clinical Trial Registry - India (CTRI/2023/08/056614).

The sample size was estimated by using the data obtained from a previous study conducted by Nagireddy VR et al. [14]. 

The sample size was computed using the following formula:

$nA = \kappa nB$ and $n_B = \left[ \frac{p_A(1 - p_A)}{\kappa} + p_B(1 - p_B) \right] \left[ \frac{(z_{1 - \alpha/2} + z_{1 - \beta})}{(p_A - p_B)^2} \right]$

where,

κ=nA/nB is the matching ratio = 1

α is Type I error = 0.05

β is Type II error, meaning 1−β is power = 95%

pA = Proportion in Group A = 0.7291

pB = Proportion in Group B = 0.34

Substituting the values from previous research, the sample size calculated was 19 per group. Considering the follow-up nature and expecting 10% drop-out rates, the final sample size was adjusted to 21 per group.

Selection of study subjects

The study included CSHCN aged 4-12 years with 3 to 4 active carious primary or permanent teeth, categorized under caries scores 3, 4, or 5 according to the ICDAS II classification. Informed consent was obtained from parents before participation. Children with caries involving pulpal exposure, fistula, swelling, history of pain, antibiotic or fluoride mouthwash usage in the past two weeks, five or more carious teeth, chronic systemic diseases affecting salivary flow, children under any ongoing drug regimen, allergies to silver or study materials, or carious teeth in the exfoliative stage were excluded.

Participants were recruited from schools for children with special needs in the Nagpur region. Children were screened based on inclusion and exclusion criteria. Eligible children were enrolled, and demographic, medical, and dental details were recorded.

Randomization and group allocation

Participants were randomly assigned to two groups using computer-generated random numbers, ensuring simple random sampling. Allocation concealment was maintained using sequentially numbered, opaque, sealed envelopes. Participants were divided into two groups: Group 1 (Control) received SDF treatment (n=21), and Group 2 (Experimental) received NSF treatment (n=21).

Single blinding was maintained during the study. Due to the cognitive limitations of the CSHCN (IQ 40-70), participants were unlikely to recognize or differentiate between the two treatment materials. Their caregivers were also not informed of the specific treatment applied. While the same clinician administered the treatment and performed the clinical evaluations, efforts were made to reduce bias by ensuring that the microbiological analysis was conducted by an independent laboratory. Salivary samples were anonymized and labeled with coded identifiers before being submitted for microbial analysis.

NSF was prepared at the Nano Research Elements Institute, Haryana, following the formulation by Nagireddy et al. [14]. The synthesis of silver nanoparticles in an aqueous solution was accomplished through the chemical reduction of silver nitrate with sodium borohydride and the use of chitosan as a stabilizing agent. First, colloidal silver was prepared by dissolving 1 g of chitosan in 200 mL of 2% (V/V) acetic acid. Thereafter, the solution was stirred overnight before being filtered under a vacuum. Following that, an aliquot of 60 mL of chitosan solution was stirred in an ice bath before being mixed with 4.0 mL of silver nitrate solution (AgNO3; 0.012 mol/L). Subsequently, sodium borohydride (NaBH4) was added to the former solution after 30 minutes. The mass ratio of AgNO3 to NaBH4 was maintained at 1:6, and the addition was done in a dropwise manner. The AgNPs formed were spherical in shape with an average size of 3.2-1.2 nm. At the end of the experiment, 0.05 ppm sodium fluoride was added to improve the stability of the solution. The concentrations of each component, expressed in micrograms per milliliter, were as follows: chitosan (28,585 g/mL), Ag+ (376.5 g/mL), and sodium fluoride (5,028.3 g/mL).

Methodology

In the first appointment, 1 ml of unstimulated saliva was collected in the morning using a sterile Pasteur pipette and transferred into a sterile Eppendorf tube. Subsequently, the application of SDF was carried out after drying the teeth and isolating them from saliva using cotton rolls. Vaseline was then applied to provide additional protection for the gingiva. However, no effort was made to remove caries or unsupported enamel. One drop of SDF was placed into a dappen dish and applied to the carious region of the teeth using a micro brush applicator tip. The SDF was left in contact with the teeth for four minutes. Any excess was removed with a cotton pellet to minimize systemic absorption. The children were instructed not to eat or drink for 30 minutes after the application, and parents were given the same instructions to avoid providing food or water during this period.

In Group 2, after saliva collection, NSF was applied similarly, with a contact time of one minute. Post-application instructions were the same as for Group 1.

The collected saliva from both groups was immediately sent to the Rajiv Gandhi Biological Technology Centre, Nagpur, for the evaluation of *Streptococcus mutans *and *lactobacilli *bacteria.

**Figure 1:**
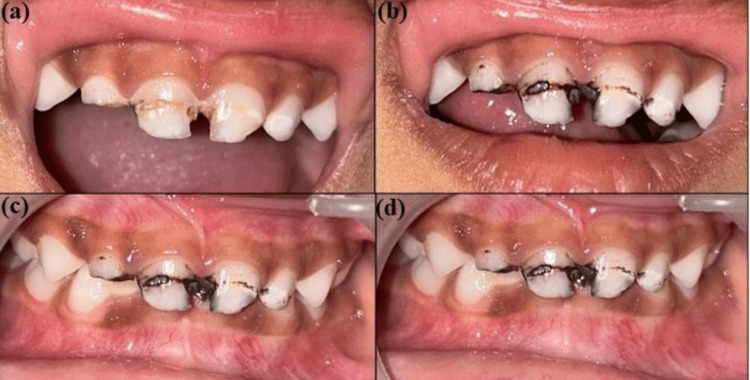
Clinical photographs before and after the application of SDF (a) Preoperative photograph with caries wrt 51, 52, 61, 62; (b) 1-month follow-up showing arrested caries wrt 51, 52, 61, 62; (c) 3-month follow-up showing arrested caries wrt 51, 52, 61, 62; (d) 6-month follow-up showing arrested caries wrt 51, 52, 61, 62 SDF: silver diamine fluoride

**Figure 2:**
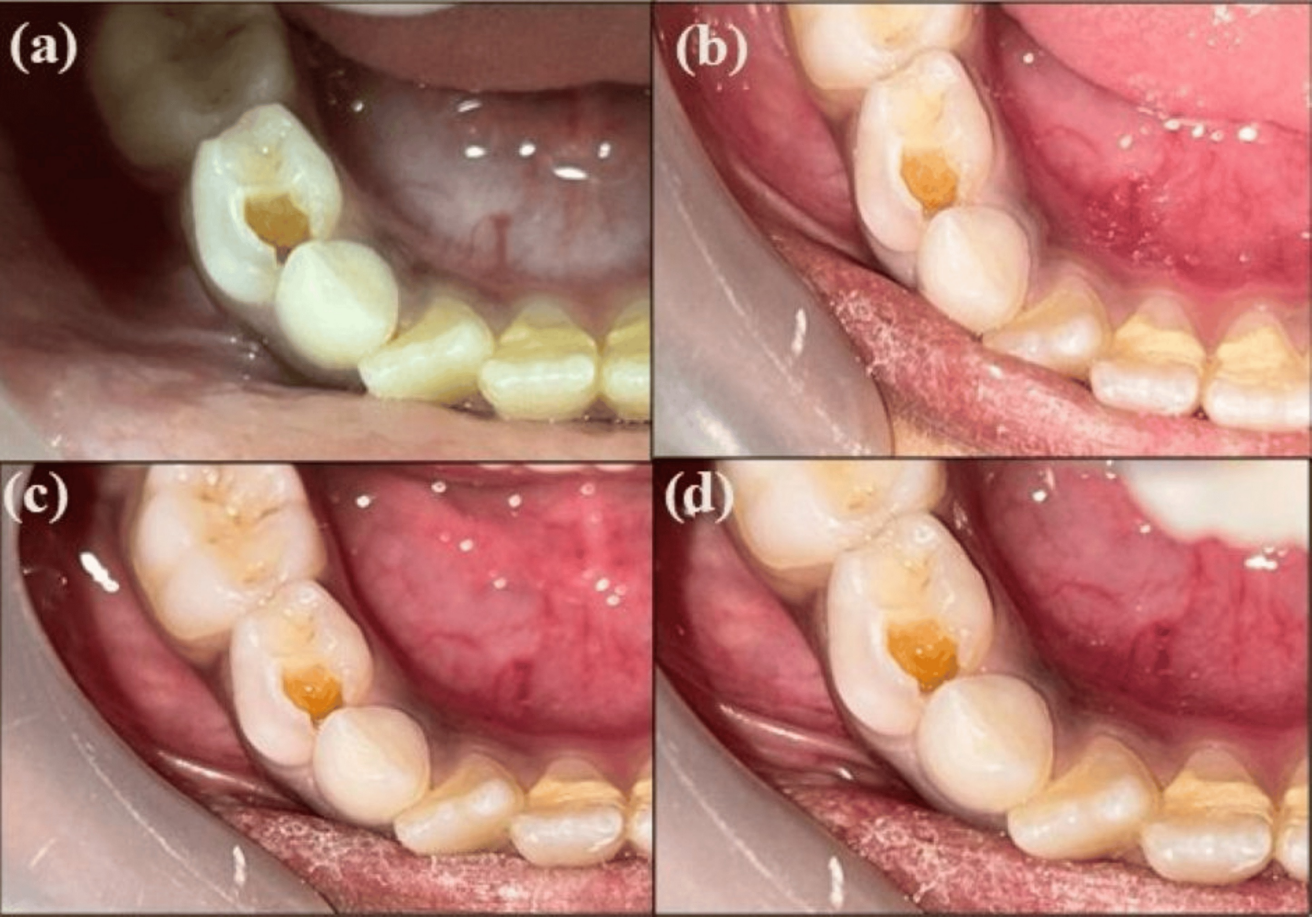
Clinical photographs before and after the application of NSF (a) Preoperative photograph with caries wrt 84; (b) 1-month follow-up showing arrested caries wrt 84; (c) 3-month follow-up showing arrested caries wrt 84; (d) 6-month follow-up showing arrested caries wrt 84 NSF: nano silver fluoride

 After one month, a follow-up was done, and 1 ml of unstimulated saliva was collected from both groups in an Eppendorf sterile tube with a pipette and was evaluated again to check the reduction count of bacteria. Moreover, arrested caries (shiny and not penetrated by the probe) were checked by the Community Periodontal Index of Treatment Needs (CPITN) probe. Three and six-month follow-ups were done only to check arrested caries (Figure 1 and Figure 2).

Microbiological procedure

Saliva samples were diluted in sterile water to achieve a 1/10,000 (10⁻⁴) dilution under laminar flow conditions. For culture preparation, 100 µL of each diluted sample was plated onto freshly prepared media. Mitis Salivarius Agar, added with bacitracin (0.2 units/mL) and 1% potassium tellurite, was used for *Streptococcus mutans*, while Rogosa agar was utilized for lactobacilli. The inoculated plates were placed inside an anaerobic jar, where an anaerobic gas pack and indicator tablets were added to maintain strict anaerobic conditions. The jar was then incubated at 37 °C for 72 hours.

**Figure 3:**
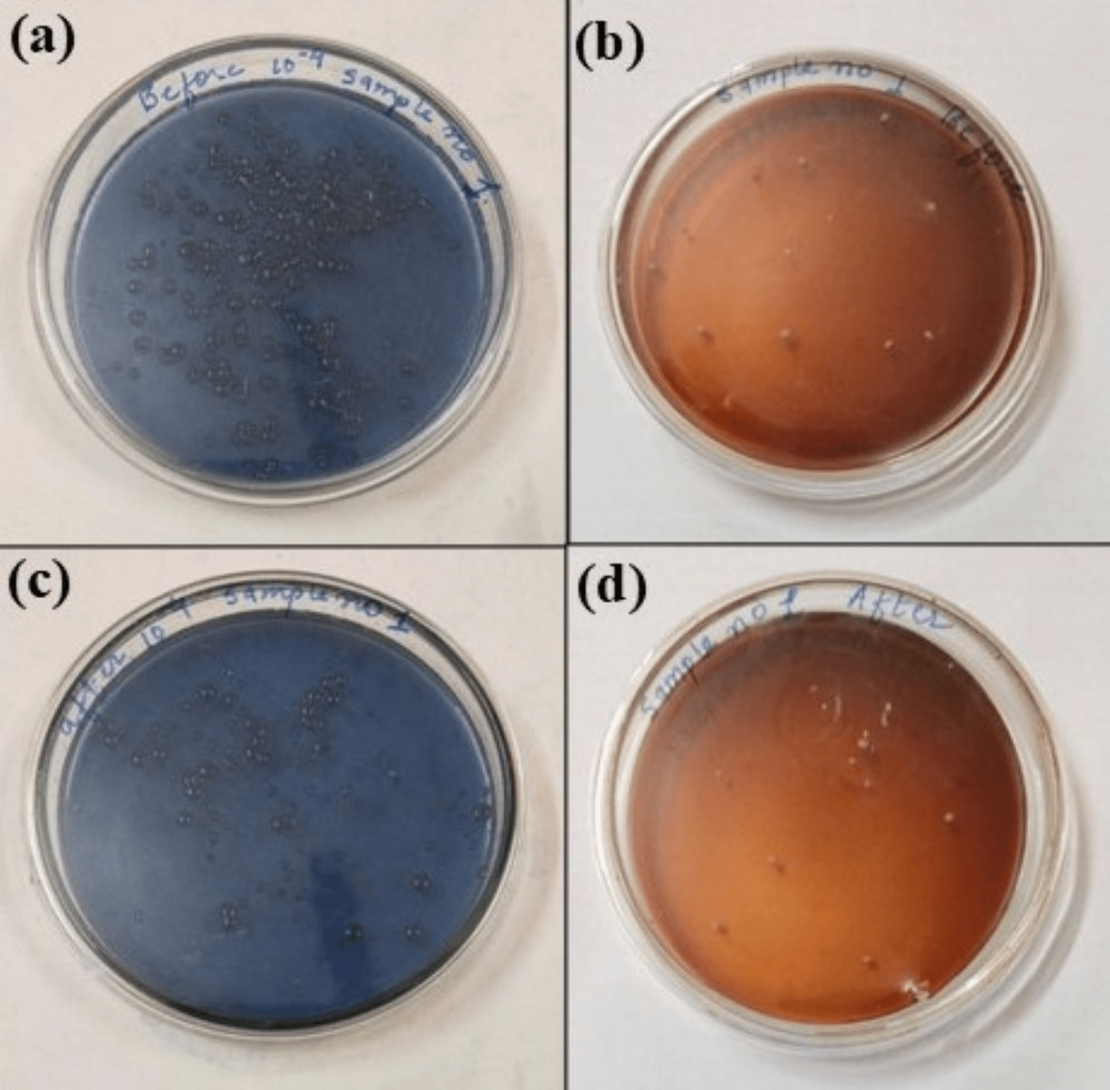
Microbiological evaluation for Streptococcus mutans and Lactobacilli in group 1 (SDF) (a) Mutans streptococci before the application of SDF; (b) Lactobacilli before the application of SDF; (c) *Streptococcus mutans* one month after the application of SDF; (d) *Lactobacilli* one month after the application of SDF SDF: silver diamine fluoride

**Figure 4:**
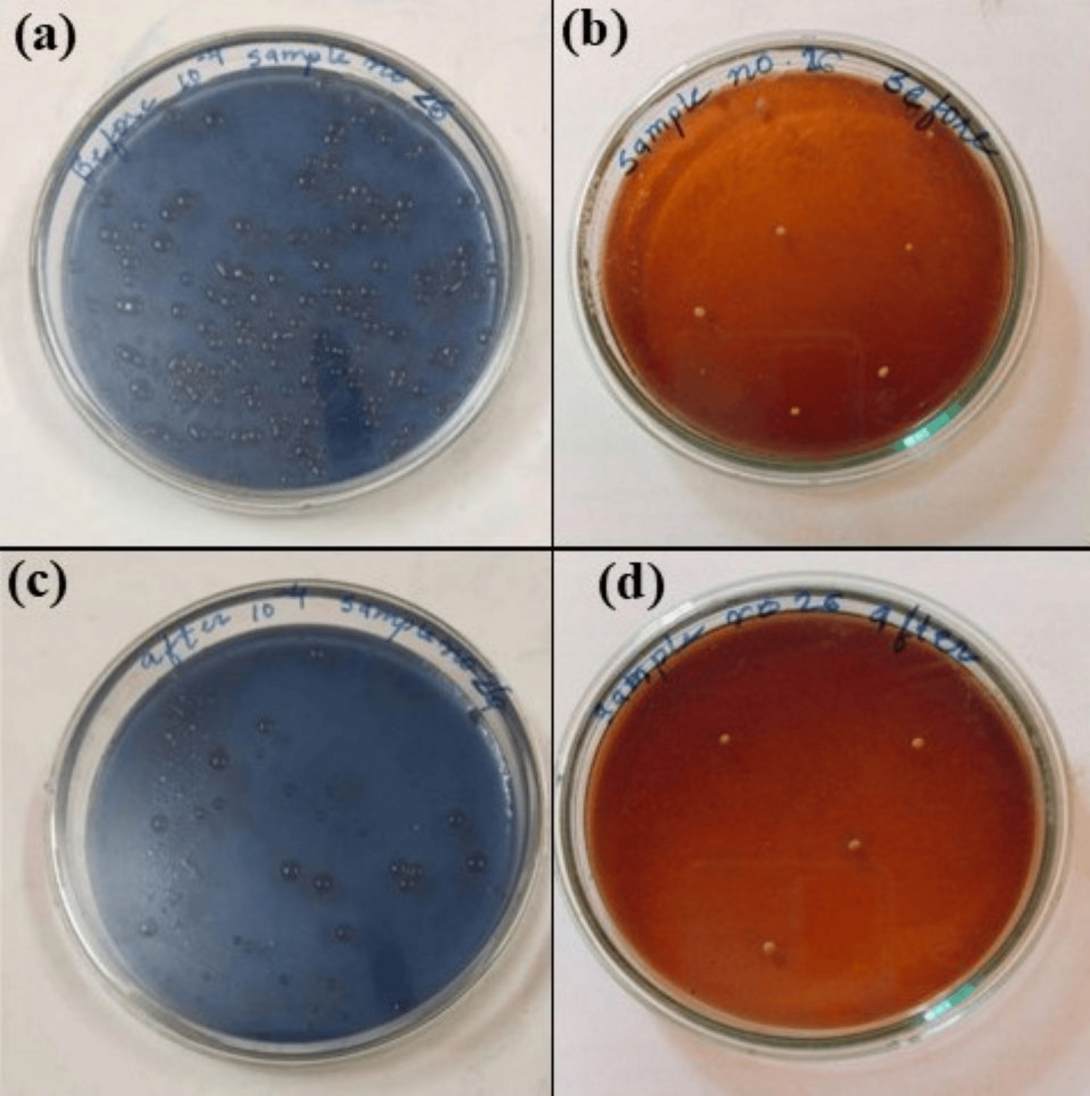
Microbiological evaluation for Streptococcus mutans and Lactobacilli in Group 2 (NSF) (a) *Streptococcus mutans* before the application of NSF; (b) *Lactobacilli *before the application of NSF; (c) *Streptococcus mutans *one month after the application of NSF; (d) *Lactobacilli *one month after the application of NSF NSF: nano silver fluoride

Following incubation, bacterial colonies of *Streptococcus mutans* and *Lactobacilli *were enumerated using a digital colony counter. The bacterial count was determined and expressed as CFU/mL, calculated using the formula: Count = Number of colonies × Reciprocal of dilution. All bacterial counts (Figure 3 and Figure 4) were documented in each child's case record form for statistical analysis.

Statistical analysis

Statistical analysis was performed using SPSS version 26.0 (IBM Corp., Armonk, NY, US). Intragroup comparisons, such as changes in active carious teeth and reductions in *Streptococcus mutans* and *Lactobacilli *counts, were analyzed using the Wilcoxon signed-rank test. Intergroup comparisons, including the number of carious, arrested, and active teeth at each follow-up and differences in bacterial count reductions, were assessed using the Mann-Whitney U test and independent t-test. A significance level of p < 0.05 was considered for all analyses.

## Results

**Figure 5:**
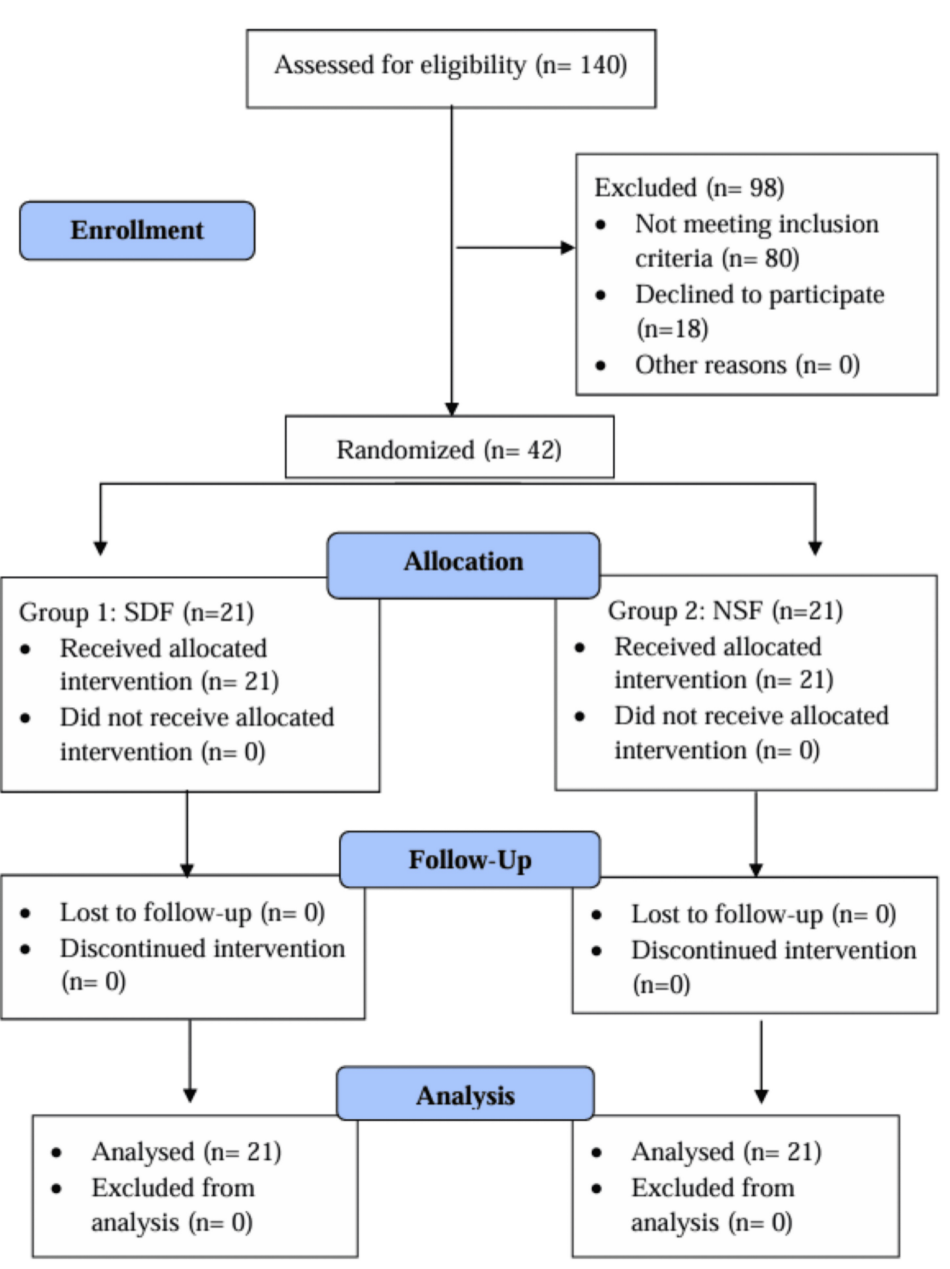
CONSORT flow diagram of the study CONSORT: Consolidated Standards of Reporting Trials

A total of 42 children participated in the study, with 21 allocated to each group. The allocation process is detailed in the CONSORT (Consolidated Standards of Reporting Trials) flow diagram (Figure 5).

The mean age of participants was 7.67 years in Group 1 and 8.05 years in Group 2. Group 1 comprised 12 males and 9 females, while Group 2 included 10 males and 11 females (Table 1).

**Table 1:** Demographic details of study participants Group 1: silver diamine fluoride, Group 2: nano silver fluoride

Group	Age in years	Gender	
		Male	Female
Group 1	7.67 ± 2.35	12 (57.1%)	9 (42.9%)
Group 2	8.05 ± 2.33	10 (47.6%)	11 (52.4%)

 The number of arrested carious teeth was compared between the two groups at 1, 3, and 6 months (Table 2).

**Table 2:** Comparison of the number of arrested carious teeth between the two groups *Mann-Whitney test Group 1: silver diamine fluoride, Group 2: nano silver fluoride

Follow-up	Teeth	Group 1	Group 2	z-value	*p-value
		Mean ± SD	Mean ± SD		
1 month	Primary	2.14 ± 1.06	2.10 ± 1.38	-0.026	0.979
	Permanent	0.86 ± 1.01	0.67 ± 0.97	-0.597	0.551
	Total	3.00 ± 0.89	2.76 ± 0.89	-0.853	0.393
3 months	Primary	2.10 ± 1.04	2.05 ± 1.12	-0.079	0.937
	Permanent	0.81 ± 0.93	0.67 ± 1.11	-0.759	0.448
	Total	2.90 ± 0.94	2.71 ± 0.78	-0.692	0.489
6 months	Primary	2.00 ± 1.14	1.95 ± 1.12	-0.157	0.876
	Permanent	0.76 ± 0.89	0.62 ± 0.97	-0.675	0.499
	Total	2.76 ± 1.09	2.57 ± 0.81	-0.837	0.403

There were no statistically significant differences in the average number of arrested primary or permanent teeth between Group 1 and Group 2 at any follow-up interval (p > 0.05). Similarly, the total number of arrested carious teeth did not differ significantly between the groups at any time point.

Within-group comparisons of active carious teeth from baseline to 6 months (Table 3) revealed a statistically significant reduction in Group 1 for both primary teeth (p < 0.001) and permanent teeth (p = 0.004).

**Table 3:** Comparison of the number of active carious teeth at baseline and after six months Wilcoxon signed-rank test * Indicates a significant difference at p≤0.05 Group 1: silver diamine fluoride, Group 2: nano silver fluoride

Group	Tooth	Baseline	6 months	z-value	p-value
		Mean ± SD	Mean ± SD		
Group 1	Primary	2.71 ± 1.19	0.71 ± 0.90	-3.861	<0.001*
	Permanent	1.10 ± 1.26	0.33 ± 0.58	-2.889	0.004*
	Total	3.81 ± 0.40	1.05 ± 1.02	-3.964	<0.001*
Group 2	Primary	2.86 ± 1.39	0.90 ± 0.83	-3.661	<0.001*
	Permanent	0.90 ± 1.34	0.29 ± 0.46	-2.588	0.010*
	Total	3.76 ± 0.44	1.19 ± 0.68	-4.084	<0.001*

Group 2 also demonstrated a significant decrease in active carious teeth over the same period (p < 0.001). When comparing the percentage of arrested teeth between groups at one, three, and six months for both primary and permanent teeth (Table 4), no statistically significant differences were observed at any time interval.

**Table 4:** Comparison of % of arrested teeth between the two groups *Mann-Whitney test Group 1: silver diamine fluoride, Group 2: nano silver fluoride

Interval	Group 1	Group 2	z-value	*p-value
	Mean ± SD	Mean ± SD		
Primary teeth				
1 month	80.83 ± 22.15	75.00 ± 22.22	-0.848	0.396
3 months	79.58 ± 23.18	71.49 ± 23.29	-0.961	0.296
6 months	76.25 ± 29.15	67.98 ± 31.09	-0.870	0.380
Permanent teeth				
1 month	78.03 ± 31.68	79.63 ± 21.29	-0.149	0.882
3 months	75.76 ± 32.80	73.15 ± 37.22	-0.073	0.941
6 months	71.21 ± 32.56	68.52 ± 32.48	-0.073	0.941

Both intergroup and intragroup comparisons of *Streptococcus mutans* counts revealed a statistically significant reduction from baseline to the one-month follow-up in both groups (p < 0.001). However, the degree of reduction in *Streptococcus mutans* counts was comparable between Group 1 and Group 2, with no significant difference observed between the groups (Table 5).

**Table 5:** Intergroup and intragroup comparison of Streptococcus mutans counts between the first appointment and the one-month follow-up * Indicates a significant difference at p≤0.05 **Independent t test, ^#^Mann-Whitney test, ^¥^Wilcoxon signed-rank test Group 1: silver diamine fluoride, Group 2: nano silver fluoride

Teeth	Group 1	Group 2	z-value	Intergroup p-value
	Mean ± SD	Mean ± SD		
1st appointment	151.57 ± 43.35	138.24 ± 32.37	-1.195	0.265**
1 month	66.52 ± 24.34	56.57 ± 29.10	-1.624	0.104^#^
z-value	-4.015	-4.016		
Intragroup p-value^¥^	<0.001*	<0.001*		

A similar pattern was noted for Lactobacilli counts, which also showed a significant decrease within each group over the same period (p < 0.001), but no significant difference in the magnitude of reduction was found between the two groups (Table 6).

**Table 6:** Intergroup and intragroup comparison of Lactobacilli counts between the first appointment and the one-month follow-up ^#^Mann-Whitney test; ^¥^Wilcoxon signed-rank test, * Indicates a significant difference at p≤0.05 Group 1: silver diamine fluoride, Group 2: nano silver fluoride

Teeth	Group 1	Group 2	z-value	Intergroup p-value^#^
	Mean ± SD	Mean ± SD		
1st appointment	17.76 ± 10.56	15.81 ± 9.86	-0.617	0.537
1 month	10.95 ± 6.67	9.48 ± 6.08	-0.694	0.488
z-value	-4.022	-4.029		
Intragroup p-value^¥^	<0.001*	<0.001*		

## Discussion

Dental caries remains a significant global health burden, particularly among CSHCN. These children often face increased challenges in maintaining oral hygiene and accessing routine dental services due to a combination of factors such as elevated treatment costs, limited cooperation, caregiver burden, and systemic barriers. Consequently, they exhibit a disproportionately high prevalence of untreated carious lesions [18]. According to the U.S. Surgeon General’s report, 45% of children aged 5-17 years are affected by dental caries, with greater severity observed among those with disabilities [19].

In response to these challenges, the present study focused on CSHCN aged 4 to 12 years with diagnosed intellectual disabilities. The included participants had IQ scores ranging from 40 to 70, classified as mild to moderate intellectual disability based on the WISC-III.

Modern caries management emphasizes prevention, early diagnosis, and minimally invasive treatments tailored to individual risk profiles. For CSHCN, such personalized approaches are essential, especially in the absence of standardized, evidence-based protocols [20]. SDF has gained attention as a non-invasive, cost-effective option for arresting cavitated lesions in children. Although effective and easy to apply, its chief limitation is the black staining of treated surfaces [21].

NSF represents a novel approach utilizing nanotechnology. It comprises silver nanoparticles, chitosan, and sodium fluoride, offering effective caries arrest without undesirable tooth discoloration [10]. This aesthetic advantage makes NSF an attractive alternative to SDF. While both agents have been studied in the general pediatric population, there is limited research on their efficacy among children with special health care needs. Therefore, the current study sought to compare the cariostatic and antibacterial effects of SDF and NSF in this specific population.

This study evaluated caries arrest rates in primary and permanent teeth at one, three, and six months post-application. In the SDF group, primary teeth showed arrest rates of 80.83%, 79.58%, and 76.25% at 1, 3, and 6 months, respectively, while permanent teeth recorded 78.03%, 75.76%, and 71.21%. The NSF group showed slightly lower arrest in primary teeth (75%, 71.49%, and 67.98%) but similar outcomes in permanent teeth (79.63%, 73.15%, and 68.52%).

Although a gradual decline in arrest rates was noted over time, the results suggest the need for reapplication at six-month intervals to maintain therapeutic effectiveness. This aligns with findings by Fung HT et al., who reported arrest rates of 79.7% and 74% in primary teeth following the biannual application of 38% SDF [22].

In addition to its cariostatic action, SDF has potent antimicrobial properties, particularly against *Streptococcus mutans* and *Lactobacilli, *key pathogens in dental caries [23]. Our findings confirmed a significant reduction in the salivary levels of these bacteria one month after applying both SDF and NSF. This is consistent with studies by Chakraborty S et al. and Alessa N et al., which highlighted the antibacterial efficacy of SDF [4,23].

Both SDF and NSF demonstrated high arrest rates in both dentitions at all follow-up points. The NSF group exhibited comparable efficacy to SDF, with no statistically significant differences, suggesting that NSF is equally effective in caries arrest. However, contrasting findings were reported by Quiritum M et al. [10], who observed higher arrest rates with NSF (78.4%) as compared to SDF (65.0%) in children under four years of age. Additionally, the absence of discoloration with NSF strengthens its clinical appeal.

The observed reduction in caries arrest over time may be attributed to factors such as biofilm reformation, dietary habits, and inconsistent oral hygiene. Despite this, both agents maintained high arrest rates at six months, underscoring their sustained efficacy. The antibacterial effects observed were consistent with those reported by Ammar N et al. [24], with significant reductions in bacterial counts post-treatment in both groups. Although intra-group changes were statistically significant, inter-group differences were not, confirming the comparable antibacterial action of SDF and NSF.

Occasional reports of a mild metallic taste were noted with NSF, although less intense than with SDF. No adverse effects, such as gingival irritation, were observed with NSF, aligning with the findings of Pushpalatha C et al. [25], who reported that NSF is biocompatible, non-staining, and non-irritating to soft tissues. Throughout the study, both agents demonstrated favorable safety profiles, reinforcing their appropriateness for use in pediatric patients with special needs.

The outcomes of this study highlight the important role of SDF and NSF in managing dental caries among CSHCN, a group with complex oral health needs. NSF, with its equivalent efficacy and superior aesthetic properties, presents a promising alternative to SDF in this population.

Nevertheless, the study's six-month follow-up period, while adequate for short-term analysis, may not fully capture the long-term stability of lesion arrest or bacterial suppression. Variables such as dietary patterns, oral hygiene practices, and caregiver involvement were not strictly controlled and may have influenced the results. Additionally, the study relied on clinical and microbiological assessments without radiographic confirmation, which could have further validated caries arrest.

Future research should address these limitations by incorporating larger and more diverse populations, extending the follow-up duration beyond one year, and including radiographic evaluations alongside clinical criteria. Moreover, exploration into the development and commercial availability of ready-to-use NSF formulations would enhance their clinical applicability.

## Conclusions

In light of the growing burden of dental caries among specially abled children, there is a compelling need to explore innovative, minimally invasive treatment modalities that not only halt disease progression but also address the aesthetic and emotional concerns associated with visible dental interventions. Children with special healthcare needs often face barriers in accessing routine dental care, making the adoption of effective, non-invasive, and patient-friendly alternatives all the more essential.

This study highlights the potential of NSF as a viable and advantageous alternative to the traditionally used SDF. While both agents share similar mechanisms in terms of their antimicrobial action and caries-arresting ability, NSF offers a crucial benefit by eliminating the characteristic black staining commonly observed with SDF. This attribute significantly enhances its appeal for use in visible areas, particularly anterior teeth, where aesthetics are often a concern for both patients and caregivers. Moreover, the favorable safety profile of NSF, coupled with its ease of application, cost-effectiveness, and acceptability, positions it as a valuable tool in preventive and therapeutic dental care for children with special needs. Its use aligns with the principles of minimally invasive dentistry, promoting effective disease control while ensuring better cooperation and satisfaction among patients and parents. In conclusion, NSF represents a promising advancement in pediatric caries management, especially for the specially abled populations. Its combination of clinical efficacy, aesthetic superiority, and safety underscores its potential for integration into both individualized dental treatment plans and large-scale community oral health initiatives. Future longitudinal studies with large samples are recommended to further validate these findings and support the broader implementation of NSF in diverse clinical settings.
